# Lens Displacement and Retinal Injury in Blunt Eye Trauma

**DOI:** 10.1155/2024/1781997

**Published:** 2024-10-22

**Authors:** Jiabei Zhou, Xinqi Ma, Fang Duan, Manli Liu, Yiyu Xie, Chongde Long

**Affiliations:** State Key Laboratory of Ophthalmology, Zhongshan Ophthalmic Center, Sun Yat-sen University, Guangdong Provincial Key Laboratory of Ophthalmology and Visual Science, Guangdong Provincial Clinical Research Center for Ocular Diseases, Guangzhou 510060, China

## Abstract

**Introduction:** We aimed to investigate the incidence and prognosis of retinal injury in patients with lens dislocation caused by blunt eye trauma.

**Methods:** We retrospectively analyzed 53 patients who underwent lensectomy and vitrectomy for contusive lens dislocation and had no preoperative retinal injuries. Patients were categorized according to the presence of retinal injury discovered intraoperatively. The clinical features of 53 eyes were assessed during a 3-month postoperative follow-up.

**Results:** Retinal injuries were observed intraoperatively in 28 patients (52.8%), predominantly in peripheral regions, with a single retinal tear being the most common type. Total lens dislocation was more frequent than subluxation in the group with retinal injuries. The intraocular pressure (IOP) at the 3-month follow-up was significantly lower than the initial IOP in both groups, with no significant differences between them. The corrected distance visual acuity (CDVA) significantly improved in both groups without significant differences.

**Conclusion:** Half of the patients without preoperative retinal injuries were found to have injuries during surgery. Total lens dislocation carried a greater risk of retinal injuries than subluxation. The improvement in CDVA after prompt retinal injury treatment did not significantly differ from that in patients without retinal injury, highlighting the importance of prompt intervention.

## 1. Introduction

Ocular trauma is a major cause of unilateral vision loss and blindness [1], with 37.8% of cases attributed to blunt eye trauma [2]. This form of injury arises from mechanical blunt force, which damages the eyeball and surrounding tissues, potentially resulting in a poor visual prognosis (blindness rate of 23.1%) [3]. Lens injuries occur in approximately 5.5% of closed globe eye injuries [4]. In lens subluxation, the breaking of zonular filaments causes the lens to lose its secure position. Lens subluxation is categorized into three types: mild (lens edge uncovers 0% to 25% of the dilated pupil), moderate (uncovers 25% to 50%), and severe (uncovers more than 50%) [5]. While patients with mild lens subluxation typically undergo phacoemulsification, pars plana lensectomy (PPL) may be considered for those with mild lens subluxation accompanied by high intraocular pressure (IOP) due to anterior movement of the subluxated lens and vitreous hernia [6]. For moderate lens subluxation, fundus ophthalmologists often opt for pars plana vitrectomy (PPV) and PPL surgeries, with comparable long-term outcomes between phacoaspiration and tension ring implantation [7]. In severe or total lens dislocation, PPV and PPL surgeries are suitable for certain cases of lens dislocation (e.g., traumatic cataracts) that may be preoperatively associated with vitreous prolapse or the risk thereof through the standard anterior approach. These procedures demonstrate that PPV and PPL surgeries lead to positive visual outcomes in complex cataract cases (lens subluxation, vitreous hernia, and iris dialysis) and provide a comprehensive retinal view during surgery [6, 8].

Blunt ocular trauma carries a risk of posterior segment tissue damage, with approximately 9% of patients experiencing retinal detachment, which significantly increases the risk of blindness [3]. Previous research indicates that in 50% of eye contusion cases with posterior lesions, these lesions were accompanied by at least one anterior segment lesion, including hyphema, iris dialysis, cataracts, or lens subluxation [9]. Posterior lesions include retinal detachment, retinal breaks without detachment, retinal or subretinal hemorrhage, retinal degeneration, and various other retinal, choroidal, and optic nerve diseases [9]. Moreover, Kuhn et al. [3] found that the incidence of retinal detachment increases to 16% when it is combined with vitreous hemorrhage. The occurrence of retinal tears can be attributed to various factors, necessitating laser or surgical interventions. In cases of blunt trauma, lens displacement often coincides with retinal tears or detachment, affecting approximately 37.7% of patients [10]. However, some fundus changes are difficult to detect before surgery; 62.2% of patients with blunt ocular trauma exhibit a cloudy refractive medium, hindering fundus observation [10]. Risk factors for low vision include retinal detachment, vitreous hemorrhage, and choroidal rupture. Among these, patients with retinal detachment have a higher proportion of postoperative low vision [3]. Identifying superficial retinal detachment, retinal hemorrhage, traumatic retinal degeneration, and simple retinal tears can be challenging during direct slit lamp examination due to cloudiness of the refractive medium. These conditions may also evade detection when using B-wave ultrasound and ultrasound biomicroscopy. Consequently, specific retinal injuries may remain undetected during preoperative examination and only become apparent during surgery. Thus, investigating the association between lens displacement and retinal injury in patients with blunt trauma is crucial, as this approach could allow the conspicuous anterior injury to serve as an indicator of underlying retinal injuries.

This study aimed to evaluate the incidence of intraoperative retinal injuries not detected preoperatively and to compare the risk of retinal injuries in patients with lens subluxation versus total dislocation. By doing so, we aim to provide valuable insights for effectively managing ocular trauma-related retinal injuries and enhancing clinical practices.

## 2. Methods

### 2.1. Study Design and Ethics

This study was approved by the Independent Institute Research Ethics Committee at Zhongshan Ophthalmic Center (ZOC), approval number (2023KYPJ262). The Institutional Review Board of the Zhongshan Ophthalmic Center waived the requirement for written informed consent for this study. All experiments were performed in accordance with the tenets of the Declaration of Helsinki.

### 2.2. Participants

A total of 53 patients with lens subluxation or total dislocation due to blunt ocular trauma who presented at the Zhongshan Ophthalmic Center, Sun Yat-sen University, between February 2020 and February 2023 were included. All enrolled patients with blunt ocular trauma presented with closed-eye injuries. The inclusion criteria were as follows: (1) Evidence of lens subluxation (within 1–11 suspensory ligament injury sites across clock hours) or total lens dislocation due to blunt ocular trauma. All patients had indications for PPV and PPL surgeries, including mild lens subluxation with high IOP caused by anterior lens movement and vitreous hernia, as well as moderate, severe, or total lens dislocation. (2) No evidence of retinal injury in preoperative evaluations. These evaluations included slit-lamp examination with fundus scanning lens (each patient underwent fundus assessment with a fundus scanning lens, and those with visible fundi underwent an extensive evaluation utilizing a three-mirror contact lens prior to the surgical procedure), fundus photography, B-ultrasound, and ultrasound biomicroscopy. (3) Surgical procedures, involving PPV and PPL, performed by a single surgeon (Professor Chongde Long) at the Zhongshan Ophthalmic Center. (4) A follow-up period of at least 3 months.

The exclusion criteria were as follows: (1) patients preoperatively diagnosed with retinal injury; (2) patients with a history of retinal tears, detachment, vitreoretinal disorders known to cause retinal rupture (e.g., hereditary retinopathy, lattice-like degeneration, degenerative retinoschisis, and acute retinal necrosis syndrome), or suspected iatrogenic retinal tears; (3) patients who underwent eye surgery before trauma (except eye adnexa surgery); and (4) patients preoperatively diagnosed with diabetic retinopathy, hypertensive retinopathy, or other retinopathies associated with systemic diseases.

Patients who met the specified inclusion and exclusion criteria were identified and subsequently enrolled in the study. The medical records of enrolled patients included demographics, medical history, corrected distance visual acuity (CDVA), IOP, slit-lamp examination (with a fundus scanning lens or three-mirror contact lens), fundus photography, ultrasound biomicroscopy, and B-ultrasound. Assessment of the CDVA was performed by proficient optometrists at the Zhongshan Ophthalmic Center, utilizing Snellen charts. IOP was calculated as an average value using a non-contact tonometer (Canon Medical System USA). All patients underwent a 23-gauge PPV combined with lensectomy. A detailed fundus examination was performed during surgery. Our postoperative follow-up period was extended to at least 3 months, with appointments scheduled for the immediate day, at 1 week, 1 month, and 3 months postsurgery. When patients could not attend the 3-month in-person visit, a designated investigator conducted a telephone follow-up arranged for CDVA and IOP evaluations at a local medical institution 3 months post-surgery using the same Snellen Chart and noncontact tonometer. Overall, 43 patients completed the 3-month follow-up, and 53 patients were involved in the final follow-up.

### 2.3. Surgery Procedure

All surgical interventions and retrobulbar anesthesia were conducted by a single proficient ophthalmologist (C.L.) with extensive experience, using a 23-gauge system, and were executed either under retrobulbar or general anesthesia. The transconjunctival incisions were meticulously placed at 3.0–3.5 mm from the limbus, using trocars at the inferotemporal, superotemporal, and superonasal quadrants. Initial steps involved anterior vitreous detachment, followed by lensectomy to remove the lens nucleus and cortex. The decision to retain the anterior capsule was contingent upon the evaluation of the extent of anterior capsule damage and lens displacement. This was succeeded by the implementation of core and peripheral vitrectomy techniques. Following this, tailored vitreoretinal procedures, contingent upon the underlying pathology, were executed. These encompassed a spectrum of interventions, including but not limited to intravitreal injections, cryocoagulation, and photocoagulation, as well as fluid/air and air/tamponade exchanges involving substances such as silicone oil and gas.

### 2.4. Statistical Analysis

Two sets of continuous data with normal distribution and equal variance were assessed using an independent sample *t*-test. For non-normal data, the Mann–Whitney *U* test was used to compare medians in two independent samples, and the paired difference Wilcoxon signed-rank test was used to compare two matched samples. Chi-square and Fisher's exact tests were used in the contingency table test. The Snellen visual acuity was transformed into logMAR visual acuity for statistical analysis, where logMAR 2, 3, and 4 corresponded to counting fingers, hand movements, and light perception, respectively. All data were analyzed using Statistical Package for the Social Sciences version 26.0 (IBM Co., NY, USA). A *p* value < 0.05 was considered statistically significant.

## 3. Results

**Figure 1:**
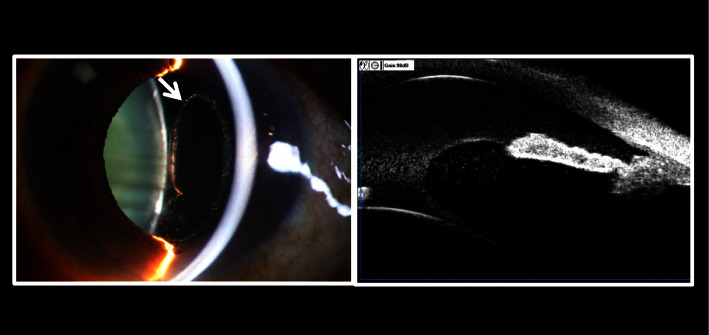
Lens subluxation and vitreous hernia (⟶).

In the 53 patients, lens subluxation or total dislocation was detected through comprehensive preoperative ocular examinations and auxiliary evaluations. However, no evidence of concurrent retinal injuries was found. Fundi were not visible in 20 of these eyes (37.7%), primarily due to corneal edema (9 eyes), hyphema (1 eye), lens opacity (9 eyes), or vitreous hernia (1 eye).  Figure 1 shows a picture of the lens subluxation and vitreous hernia. All 53 patients underwent a 23-gauge vitrectomy and lensectomy. Patients were divided into two groups based on the presence of retinal injuries discovered during surgery for retrospective analysis.  Table 1 presents the patient demographics, the duration from blunt injury to arrival at Zhongshan Ophthalmic Center for surgery, and preoperative CDVA. The patients consisted of 43 men (81.1%) and 10 women (18.9%), with an average age of 52.4 years (range 4–78 years, median 55 years). No significant differences in sex ratio or age were observed between the two groups. The mean duration between the blunt injury and the arrival at Zhongshan Ophthalmic Center for surgery was 14.39 months (range, 0 days to 31 years; median, 0.4 months). Ten eyes had undergone emergency surgeries due to trauma before PPV and PPL surgeries, including eyelid sutures in six cases, anterior chamber irrigation in two cases, conjunctival laceration suture in one case, and lacrimal canal implantation in one case. No statistically significant difference in initial CDVA was observed between the two groups (*p*=0.496).

Table 2 presents the causes of blunt trauma among patients. The most common cause of blunt ocular trauma was branch/bamboo injuries, which affected 11 eyes (20.8%). This was followed by road accidents/falls in 10 eyes (18.9%) and workplace metal/nail injuries in 9 eyes (17.0%). Other causes included domestic items (11.3%), rebound injuries (9.4%), badminton accidents (9.4%), stone injuries (5.7%), firecracker/explosive injuries (5.7%), and hand/fist injuries (1.9%).

**Figure 2:**
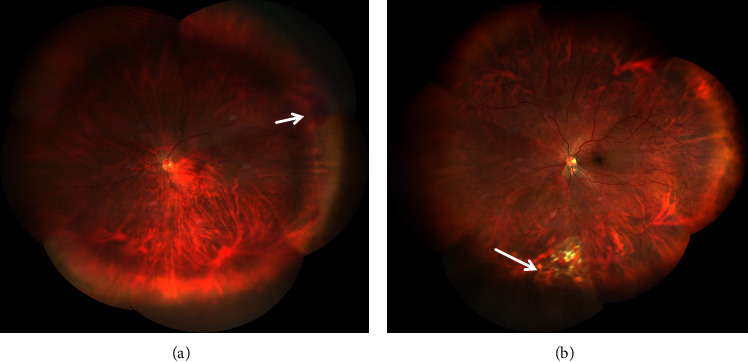
Retinal injuries, including retinal bleeding (a) and retinal tear (b). Images were collected at the first follow-up examination after pars plana vitrectomy and pars plana lensectomy surgery.

During the surgical procedure, previously undetected retinal injuries were identified in 28 of the 53 eyes (52.8%). Among these 28 patients, four with retinal detachment underwent silicone oil tamponade, while two received sterilized air tamponade. The remaining patients underwent filtration using a Bss Sterile irrigating solution. Additionally, intraocular lens implantation (Sensar AR40e, Bausch + Lomb., USA) was conducted via transscleral suture fixation in three cases without vitreous hemorrhage or retinal tears.  Figure 2 shows retinal injuries, including retinal bleeding (a) and tears (b) in peripheral areas, as observed at the first follow-up after surgery.

In patients with lens subluxation, the average number of suspensory ligament rupture sites (clock hours) in those with retinal injuries discovered intraoperatively was 6.2 ± 2.8 (range 1–11). In patients with subluxation but without any retinal injuries observed intraoperatively, the mean number of suspensory ligament rupture sites (clock hours) was 6.0 ± 1.8 (range 1–9). However, no significant difference was observed between the two groups (*p*=0.845). In patients with lens subluxation, no statistically significant association was observed between suspensory ligament injury sites (clock hours) and the presence or absence of retinal injuries (*p*=0.852).

Among the 53 eyes, 30 (56.6%) had lens subluxation, and 23 (43.4%) had total lens dislocation. The retinal damage rate in patients with lens subluxation was 40%, and the retinal injury rate in patients with total lens dislocation was 69.6%. Moreover, a statistical difference in the retinal injury rate was observed between the two groups, with total lens dislocation associated with a higher risk of retinal injury compared to lens subluxation, as shown in Table 3.

A total of 28 eyes with traumatic lens subluxation or dislocation experienced 42 retinal injuries, categorized as follows: 9 (32.1%) had a single retinal tear, 6 (21.4%) had multiple retinal tears, 5 (17.8%) had an intraretinal or a subretinal hemorrhage, 15 (53.6%) had traumatic retinal degeneration, and 7 (25%) had retinal detachment. The locations of the 42 retinal injuries were as follows: 30 (71.4%) in the far periphery, 3 (7.1%) in the mid-periphery, 2 (4.8%) in the posterior area, 6 (14.3%) in the ora serrata, and 1 (2.4%) in the pan-retina. The injuries in peripheral area, encompassing the far periphery and mid-periphery, accounted for 33 cases (78.5%).

Among all patients, 22 eyes (41.5%) had preoperative IOP levels > 21 mmHg. The postoperative changes in the mean IOP at the 3-month follow-up are shown in Table 4. Overall, 39 patients completed the 3-month follow-up (18 patients in the group with retinal injuries and 21 patients in the group without retinal injuries). A significant decrease in IOP from the initial visit to the 3-month follow-up was observed in the nonretinal injury group (*p*=0.016). However, no significant differences were observed neither within the retinal injury group nor between the two groups.

The postoperative changes in mean CDVA at the 3-month follow-up are shown in Table 5. Overall, 43 patients completed the 3-month follow-up (22 patients in the group with retinal injuries and 21 patients in the group without retinal injuries). CDVA improved significantly in both groups postoperatively (*p* < 0.05), and there was no significant difference between the two groups. The mean follow-up duration was 194 days (range, 90–715 days). All of the 53 eyes included in the study completed the last follow-up, of which 46 (86.8%) showed improved CDVA compared to the initial visit.

## 4. Discussion

Blunt ocular trauma constitutes approximately one-third of all ocular trauma cases [11]. In our study, blunt ocular trauma showed a sex preference with a male predominance (43 out of 53, 81.1%). This finding closely aligns with the results of a previously reported study on ocular trauma incidence, which indicated a higher occurrence of ocular trauma in men (76.1%) [12]. A total of 10 eyes underwent emergency surgery prior to the main procedure, including 8 external eye surgeries and 2 anterior chamber irrigations by experienced surgeons at Zhongshan Eye Center. No retinal damage was observed in these 2 cases. We believe the emergency surgeries are unrelated to the retinal damage seen during the primary procedure. The leading cause of blunt ocular trauma in our study was attributed to branch/bamboo injuries, affecting 11 eyes (20.8%). This cause is similar to that reported in another study on contusive lens dislocation, which identified wood chopping as a common cause (31.10%) [10].

Earlier research has demonstrated that 37.7% of patients with contusive lens displacement exhibit accompanying retinal rupture or detachment, often compounded by refractive interstitial opacity [10]. Due to interstitial opacity induced by corneal edema, anterior chamber hyphema, cataract, or vitreous hemorrhage, only a few cases of retinal damage (8.9%) are identified before surgery [10]. In our study, 28 out of 53 eyes were found to have retinal damage during surgery. Among the patients, 20 presented with an invisible fundus due to refractive medium opacity before surgery; of these, 9 (45%) had retinal injuries.

The peripheral retina, supplied by a single layer of capillaries, is susceptible to degeneration and atrophy, making it prone to retinal injuries [13–15]. A previous study showed that breaks in peripheral areas (63.6%) were found more frequently than in other regions [10]. In our study, a total of 42 retinal injuries were identified in 28 eyes, primarily in the peripheral regions. The presence of refractive medium opacity and the preferential location of retinal damage in the peripheral region render preoperative retinal damage detection challenging.

Lens displacement is common in cases of blunt ocular trauma due to its low energy threshold for causing injury compared with other tissues, combined with the presence of a thin capsule (2-3 *μ*m) and suspensory ligament (0.35–1.0 *μ*m) [16]. Our study identified the vitreous cavity (28.5%), inferior nasal region (17.8%), and inferior temporal region (14.2%) as the most common preoperative locations of lens displacement. This is consistent with previous studies that found that areas prone to suspensory ligament injuries are the superior temporal and superior nasal regions [17]. The peripheral area was most frequently affected by retinal injury (78.5%). Retinal tears in patients with eye injury are common in the upper and lower temporal quadrants [18]. Inferior temporal and upper nasal retinas are susceptible to retinal tears in patients with blunt eye trauma [19]. Factors contributing to this vulnerability include exposed subtemporal sclera, lack of eyelid protection, the Bell phenomenon causing upward and outward eyeball rotation when the eyelid is closed, poor blood supply, thin nerve fibers on the temporal side of the peripheral retina, cystic degeneration, and localized vitreous adhesion in the periphery of the inferior temporal quadrant [20, 21]. In our study, retinal injuries detected during surgery were common in the superior temporal (19%) and superior nasal areas (23.8%), which coincided with the preoperative direction of lens displacement (superior temporal [14.2%] and superior nasal [17.8%] areas). However, expanding the sample size for further research is necessary.

In our study, the prevalence of total lens dislocation with intraoperative retinal injury was higher than that with lens subluxation (66.6% vs. 43.7%). Finite element model studies of the human eye have found that when the eye is in dynamic deformation due to decompression, negative pressure and relative inertial movement may pull the retina away from the supporting tissue [22]. Vitreous basal avulsion is a more common feature of retinal detachment in patients with ocular contusion [19]. Lens dislocation is observed across the full range of impact velocities in the ocular contusion model, most commonly backward (into the vitreous); however, at higher velocities, the lens occasionally bounces back and remains in the anterior chamber [23]. Dislocations are often accompanied by rupture of the lens capsule and small-band fibers. We speculate that total lens dislocation into the vitreous cavity exerts a greater force on Wieger's ligament and the anterior hyaloid during injury compared to lens subluxation, resulting in vitreous traction on the retina. This may be the reason why patients with total dislocation are more likely to develop retinal damage [19]; however, further experiments are needed. Our research suggests that lens subluxation can also lead to retinal damage for reasons that are not yet understood. Mangan et al. [24] found that an anterior capsule rupture, compared to a posterior capsule rupture, is associated with a lower possibility of retinal injury. This is because the rupture of the posterior capsule results from the direct impact force, whereas the rupture of the anterior capsule may have a protective effect on the retina due to the countercoup effect. Therefore, in clinical practice, special attention should be paid to fundus injury in patients with posterior capsule rupture. Further investigation into the role of injury sites, such as the anterior and posterior capsules, on retinal injury is warranted [24, 25]. These factors may have contributed to the higher incidence of retinal injuries observed in patients with total lens dislocation. Patients with subluxation ranged from 1–11 clock hours, the incidence of which was not statistically significant in the presence or absence of retinal injury.

In the group with or without retinal injury, the 3-month follow-up IOP decreased significantly compared with the preoperative IOP. Considering that studies have shown that IOP significantly increases from baseline in patients who undergo vitrectomy with silicone oil tamponade, we excluded the IOP readings of patients with silicone oil tamponade from our calculations [26]. A cohort study reported that patients with trauma-related glaucoma experienced decreased IOP at the final follow-up after undergoing surgeries [27]. Watts et al. [28] also reported a lowered risk of glaucoma when vitrectomy and lensectomy were performed simultaneously for posterior dislocation of the lens. The results of our study are consistent with those of previous studies. A significant improvement in CDVA was observed 3 months after surgery in both groups compared to preoperative CDVA. However, our study found no significant difference in CDVA between the two groups (*p*=0.550), highlighting the importance of timely identification and treatment of retinal injuries during surgery. Previous studies have shown that patients with retinal injuries due to blunt eye trauma generally experience improved vision after fundus surgery [10, 29]. This is consistent with the results of our study.

The limitations of this study include the relatively low number of patients, its retrospective nature, and potential biases in some cases. However, thorough preoperative evaluations and precise surgical techniques would add to the study's credibility. Subsequent investigations might consider employing prospective study designs and increasing participant cohorts to delve into the intricate mechanisms underlying the heightened susceptibility to retinal impairment observed in individuals afflicted with complete lens dislocation. Moreover, a comprehensive examination of long-term visual outcomes could provide valuable insights.

Our study indicates that 52.8% of patients with lens displacement following blunt eye trauma, who had no evidence of retinal injuries during preoperative evaluations, were found to have retinal injuries during surgery. Total lens dislocation was associated with a higher risk of retinal injury compared to lens subluxation. The improvement in CDVA following timely treatment of retinal injury did not significantly differ from that observed in patients without retinal injury, emphasizing the importance of prompt intervention.

## Figures and Tables

**Table 1:** Demographics and clinical characteristics of patients with traumatic lens displacement.

Characteristic	Total (*n* = 53)	With retinal injuries (*n* = 28)	Without retinal injuries (*n* = 25)	*p*
Age (year)	52.4 ± 14.3 (range 4–78)	51.1 ± 14.3	53.8 ± 14.4	0.504^a^
Sex				1^b^
Male (%)	43 (81.1%)	23 (82.1%)	20 (80%)	
Female (%)	10 (18.9%)	5 (17.9%)	5 (20%)	
Time interval from blunt injury to the arrival at ZOC for surgery (month)	14.39 ± 60.07 (median 0.40, range 0–372)	13.83 ± 70.20	15.03 ± 50.36	0.284^c^
Initial CDVA (logMAR)	1.6 ± 1.0 (range 0–3.0)	1.4 ± 1.1	1.1 ± 0.8	0.496^c^

Abbreviation: CDVA, corrected distance visual acuity.

^a^Independent *t* test.

^b^Continuity correction.

^c^Mann–Whitney *U* test.

**Table 2:** Causes of blunt trauma in patients with traumatic lens displacement.

Reason for blunt trauma	Total (*n* = 53)	With retinal injuries (*n* = 28)	Without retinal injuries (*n* = 25)	*p* ∗
Firecracker/explosive injury	3	1	2	0.714
Accident/falls	10	5	5	
Metal/nail in the workplace	9	6	3	
Domestic items	6	4	2	
Stone	3	1	2	
Hand/fist	1	0	1	
Branch/bamboo	11	7	4	
Rebound injury	5	1	4	
Badminton accident	5	3	2	

^∗^Fisher's exact test.

**Table 3:** Lens displacement and retinal injury.

Lens displacement	Retinal injury		*p* ∗
	With	Without	
Lens subluxation	12	18	0.033
Total lens dislocation	16	7	

^∗^Chi square test.

**Table 4:** Changes in mean intraocular pressure (IOP).

	With retinal injuries			Without retinal injuries		
	Initial	3-month follow-up	*p* ∗	Initial	3-month follow-up	*p* ∗
IOP (mmHg)	20.3 ± 12.0	13.3 ± 2.6	0.048	20.7 ± 12.4	14.4 ± 3.9	0.016

^∗^Wilcoxon signed ranks test.

**Table 5:** Changes in mean corrected distance visual acuity (CDVA).

	With retinal injuries			Without retinal injuries		
	Initial	3-month follow-up	*p* ∗	Initial	3-month follow-up	*p* ∗
CDVA (logMAR)	1.4 ± 1.1	1.1 ± 1.0	0.046	1.1 ± 0.8	0.7 ± 0.7	0.048

^∗^Wilcoxon signed ranks test.

## Data Availability

The data are not publicly available due to ethical reasons. Further inquiries can be directed to the corresponding author.
